# Vitamin D supplementation for treatment of seasonal affective symptoms in healthcare professionals: a double-blind randomised placebo-controlled trial

**DOI:** 10.1186/1756-0500-7-528

**Published:** 2014-08-14

**Authors:** Tenna Bloch Frandsen, Manan Pareek, Jens Peter Hansen, Connie Thuroee Nielsen

**Affiliations:** Department of Mental Health Services Esbjerg, Gl. Vardevej 101, 6715 Esbjerg N, Denmark; Unit for Health Promotion Research, University of Southern Denmark, Niels Bohrs Vej 9-10, 6700 Esbjerg, Denmark

**Keywords:** Seasonal affective disorder, Vitamin D, Health personnel, Randomised controlled trial

## Abstract

**Background:**

Low serum 25-hydroxyvitamin D levels (25(OH)D) have been associated with a higher likelihood of seasonal affective disorder (SAD) and poor mental well-being, yet firm evidence for either remains lacking. Thus, vitamin D supplementation may alleviate symptoms associated with SAD.

**Methods:**

This study was a randomized, single-centre, double-blind, placebo-controlled trial including healthcare professionals employed in psychiatric and somatic hospitals. 3345 healthcare professionals were invited to participate, 50 participants were screened, and 34 were able to complete the study. The main inclusion criterion was 8 points or more on question no. 2 of the *Seasonal Pattern Assessment Questionnaire* (SPAQ-SAD). During a 3-month period, the participants received a daily dose of 70 μg vitamin D or placebo. The primary outcome was the sum of the self-reported questionnaire *Structured Interview Guide for the Hamilton Depression Rating Scale, Seasonal Affective Disorders* (SIGH-SAD). The secondary outcome was World Health Organization-Five Well-Being Index (WHO-5) of the healthcare professionals during the winter period and the exploratory outcome measures were weight, waist circumference, blood pressure, absenteeism from work and 25(OH)D.

**Results:**

There were no significant between-group differences in SIGH–SAD sums at 12 weeks (p = 0.7 (CI: - 3.27 to 4.81)). However, there was a significant improvement of primary SIGH-SAD over time from inclusion (autumn-winter) to the completion of the study (winter-spring) for all participants. The secondary and exploratory outcome measures were all insignificant between groups.

The sums of the SIGH–SAD at 12 weeks were not significantly different [*p* = 0.701 (CI: 4.81–3.27)] between the groups. There was, however, a significant improvement in primary SIGH-SAD sums over time from inclusion (autumn-winter) to the completion of the study (winter-spring) in both groups. The secondary and explorative outcome measures were not significantly different between groups.

**Conclusions:**

There were no significant between-group differences in the primary (SIGH-SAD) and secondary (WH0-5) as well as the exploratory outcome measures (weight, waist circumference, blood pressure, absenteeism from work and 25(OH)D. Thus, the study failed to demonstrate an effect of vitamin D on SAD symptoms, but our findings may be limited by confounders. Furthermore, the study was underpowered and did not allow us to assess the ability of vitamin D to improve mood in those with low 25(OH)D.

**Trial registration:**

( http://www.clinicaltrials.gov registration number: NCT01462058).

## Background

Seasonal affective disorder (SAD) [1] manifests as a clinical syndrome with symptoms of depression, carbohydrate craving, hypersomnia, lethargy and changes in circadian rhythms. The symptoms recur each winter and disappear again during spring or early summer. Women are more affected than men, and the prevalence at high latitudes ranges from 1 to 10% [2]. It has been suggested that the seasonality and symptoms of SAD may be due to low levels of serum 25-hydroxyvitamin D levels (25(OH)D) [3]. In the brain 1 alpha-hydroxylase converts 25-hydroxyvitamin D to the active vitamin D (1,25(OH)D). Further vitamin D receptors (VDR) are widespread in the human brain. Thus, it is proposed that vitamin D operates as a neurosteroid and thought to be involved in the pathogenesis of depression [3, 4].

Ultraviolet B shortwave (UVB) irradiation of the skin is the most important source of vitamin D. Irradiation on a summer day between 20–30 minutes on the face and arms results in the synthesis of 100–250 μg of vitamin D, which is subsequently activated in the liver to 25-hydroxyvitamin D. Solar UVB content at high latitudes (above 37 N) is minimal between October and March, and consequently, the synthesis and stores of vitamin D during winter and early spring are low [5]. In Denmark, suboptimal 25(OH)D status is common; thus, the prevalence of vitamin D deficiency among blood donors is 18%, whereas 47% have vitamin D insufficiency [6]. Normal 25(OH)D vary between 50–160 nmol/L, whereas values between 25–50 nmol/L are defined as vitamin D insufficiency, and deficiency is defined as less than 25 nmol/L. Additionally, 25(OH)D are lower in persons with darker skin (types III–VI) as assessed by the Fitzpatrick classification [7, 8].

Vitamin D supplementation in relation to well-being during the winter season has been studied in three small studies. A positive effect on well-being and symptoms of depression was demonstrated, when high doses of vitamin D (≥100 μg D3 daily) were given for 1 to 3 months [9–11]. One large study (n = 441) demonstrated similar significant improvement in *Beck Depression Inventory* (BDI) scores in treatment groups receiving 70 μg and 140 μg compared to placebo during a 1 year period [12]. Other large studies have failed to demonstrate effects on symptoms of depression when low dose vitamin D (≤20 μg daily) was given [13, 14].

Indoor work has been hypothesized as a risk factor for mood difficulties among civil servants in a Danish cross-sectional study (latitude 55-57N°), a significant risk reduction (odds ratio = 0.63) being found in outdoor workers (>2 hours/day) [15]. Thus, indoor work might be a significant risk factor for SAD, since indoor work is by far the most prevalent form of work among the total work force at temperate latitudes [16].

The most efficient and well-documented therapy for SAD is light therapy (LT) [17]. However, the neurobiological impact and mechanism of LT is not fully understood. SAD was at first believed to be related to abnormal melatonin metabolism, but later findings did not support this hypothesis. Thus, LT during two weeks can reduce subjective sleepiness, but the effect is not associated with a rise in levels of melatonin in saliva or 25(OH)D [18]. However, LT can be challenging to apply, and compliance might be reduced due to lack of time and social support [19, 20]. Tablet treatment can therefore be attractive for some SAD patients. Although studies of brain serotonin function support the hypothesis of disturbed activity [20], there is still too little evidence to draw any overall conclusion regarding the use of second generation antidepressants for SAD [21]. Bases on estimates of high prevalence of vitamin D insufficiency in the general Danish population and increased mood difficulties among Danish indoor compared to outdoor working personnel: vitamin D supplementation might be a solution. Thus it could be anticipated that a majority of the participants with known symptoms of SAD would develop vitamin D insufficiency during wintertime and thereby benefit from vitamin D supplementation, which in the above-mentioned winter season studies has shown positive effect on well-being.

The purpose of this study was to examine whether vitamin D supplementation would reduce SAD symptoms among indoor workers who had experienced winter depressive symptoms in the past. The secondary objective was to examine correlations between 25(OH)D, SAD and mental well-being.

## Methods

### Design

The study was a randomised, single-centre, double-blind, placebo-controlled trial including healthcare professionals employed in psychiatric and somatic hospitals. The participants were suffering from moderate seasonality with SAD symptoms [22]. The participants were randomized to either 70 μg vitamin D or placebo for a period of 12 weeks during the winter period. The dose of 70 μg vitamin D was chosen in accordance with the previous study by Jorde et al. [12] in which the lowest dose of two active drug arms (70 μg or 140 μg) significant improved BDI, without significant differences in side effects as compared to the placebo group.

### The setting

Healthcare professionals were recruited from somatic and psychiatric hospitals in the Region of Southern Denmark. The study was carried out by The Research Unit, Department of Mental Health Services, Esbjerg, during the period from October 1 2011 to March 31 2012.

### The study population

Healthcare professionals working in one of the above-mentioned departments were invited to participate in the study via direct e-mail, flyers and posters. If the invited healthcare professionals wished to participate, information were sent to them along with the Danish version of the SAD questionnaire, Seasonal Pattern Assessment Questionnaire (SPAQ-SAD) [23]. SPAQ-SAD is validated in both psychiatric patients and healthy individuals, and question 2 (The Seasonality Score) in the Danish version is the most important and best studied item of SPAQ-SAD. In the Seasonality Score, the individuals report the effect the changing of the seasons has on them in six areas (sleep length, social activity, mood, weight, appetite and energy level) on a scale of 0 to 4, with 0 being "no change" and 4 being "extremely marked change" [23]. The test-retest correlation for Seasonality Score ranges from 0.65 to 0.87, and the Seasonality Score has a good internal consistency, Cronbach’s alpha = 0.82 [24]. We decided to invite participants for a baseline visit if they rated 8 or more in question 2 in the SPAQ-SAD questionnaire. We chose a cut-off at 8 in order to include enough participants and gain power; thus, we included participants with less severe symptoms equivalent to moderate seasonality with SAD symptoms. Thus we did not use the recommended definition by Kasper et al. [22]. Diagnostic interviews were not planned due to the public health perspective focusing on symptoms of SAD rather than the full SAD diagnosis. Participants were eligible if they were 18–65 years old and had signed a written informed consent form. Exclusion criteria were any form of schizophrenia, bipolar affective disorder, sarcoidosis, tuberculosis and pregnancy, or an intake of more than 10 μg vitamin D per day or allergy to the content of the pills. Women of childbearing potential were required to use effective contraception, and a negative human chorionic gonadotropin (HCG) pregnancy test for each female participant was also required at baseline. Furthermore, participants were excluded if they had a baseline serum 25(OH)D < 10 nmol/L or > 160 nmol/L, serum calcium > 1.40 nmol/L, serum phosphate < 1.50 nmol/L (females) or < 1.60 nmol/L (males aged 18–49 years) and <1.35 nmol/l (males > 49 years), estimated glomerular filtration rate (eGFR) < 60 mL/min/1.73 m^2^ or serum parathyroid hormone (PTH) > 9.2 pmol/L.

Eligible participants received information on the study by the research nurse. The study drug was handed out along with instructions regarding the blood samples after informed consent was obtained. A consultant psychiatrist determined whether all inclusion criteria and no exclusion criteria had been met and enrolled the participants.

### Randomisation and blinding

The tablets were produced by Vimenco, Denmark, and were identical in size, smell and taste. The participants were randomised using blocks of four into the intervention group or the control group. The allocation sequence was computer generated by the Hospital Pharmacy Funen, Denmark during the labelling procedure and was concealed for staff and researchers involved in the trial.

### Outcome variables

#### Primary outcome

The primary outcome was the sum of the Danish version of the validated self-reported questionnaire *Structured Interview Guide for the Hamilton Depression Rating Scale, Seasonal Affective Disorders* (SIGH-SAD) [25].

### Secondary outcome and explorative outcome measures

Secondary outcomes were the sum of the Danish version of the validated self-reported *World Health Organization-Five Well-Being Index* (WHO-5) [26]. Weight, waist circumference, blood pressure, absenteeism from work and 25(OH)D were exploratory outcome measures.

### Assessments

The primary outcome was rated at each visit using a 24-item SIGH-SAD, a scripted version of the Hamilton Depression Rating Scale [27] modified to better reflect the atypical symptomatology of SAD [25]. This version of the SIGH-SAD consists of the HDRS 17-item scale plus the first 7 atypical items (i.e. excluding reverse diurnal variation). The Danish version was translated from English and back-translated under supervision by Claus Martiny PhD, Psychiatric Research Unit, Frederiksborg General Hospital.

Primary, secondary and explorative outcomes were assessed at baseline and at 12 weeks after baseline. Additionally, skin type and socio-demographic parameters were recorded at baseline. Known side effects of vitamin D supplementation, e.g. fatigue, muscle spasm, pain, nausea and constipation as well as severe and adverse events were systematically recorded yes or no at baseline and endpoint. The use of nutritional supplements and medication including vitamin D was recorded at baseline and at the 12-week follow-up.

Treatment adherence was assessed by the counting the amount of tablets that the participants returned at 12 weeks.

Blood samples were collected at baseline and 12 weeks later. The blood was analysed for C-reactive protein (CRP), ionised calcium, phosphate, creatinine, eGFR, PTH and 25(OH)D (to be reported only at the end of the trial). PTH analyses were performed on Immulite 2000 from Siemens using a 2-sided chemiluminescent enzyme-labelled immunometric method. 25(OH)D were analysed on ISYS equipment from IDS-Nordic, which measures the total amount of vitamin D2 and D3.

### Statistical analyses

Differences between the intervention and control groups with regard to demographic and clinical characteristics at baseline were tested using Fisher’s exact test for categorical variables and Wilcoxon rank sum test for numerical variables. For each group, the mean SIGH-SAD level was calculated at baseline and after 12 weeks. The difference between the two groups with regard to increase of SIGH-SAD sums over time was compared using the Wilcoxon rank sum test. In addition, differences between the two groups in each of the secondary and exploratory outcomes were tested using Wilcoxon rank sum test. Correlations between 25(OH)D and SIGH-SAD and WHO-5 were measured using Spearman correlation coefficients. The analyses were conducted according to intention to treat principles.

All analyses were conducted using STATA/IC 11 (StataCorp LP, College Station, Texas, USA).

### Power calculation

We expected a mean reduction of 3 points (standard deviation (SD) 6) on the SIGH-SAD according to the smallest clinical important difference in the primary outcome variable, consistent with a clinical effect of 0.5. Assuming an alpha of 0.05 and a power of 0.90, a sample size of 85 in each group was calculated. However, only 43 were randomised because of exclusion criteria and few responders. Only 34 completed the study after drop out, i.e. 20% of the number needed to give meaningful results according the power calculation. The achieved sample size reduced the opportunity to measure a difference of 6 on the scale used.

### Ethical approval

The study was approved by the Danish Data Protection Agency, the Ethics Committee for the Region of Southern Denmark (ID 301115) and the National Board of Health (Eudract: 2011-002585-20). The complete protocol can be acquired by contacting the corresponding author.

## Results

### Participant flow

**Figure 1:**
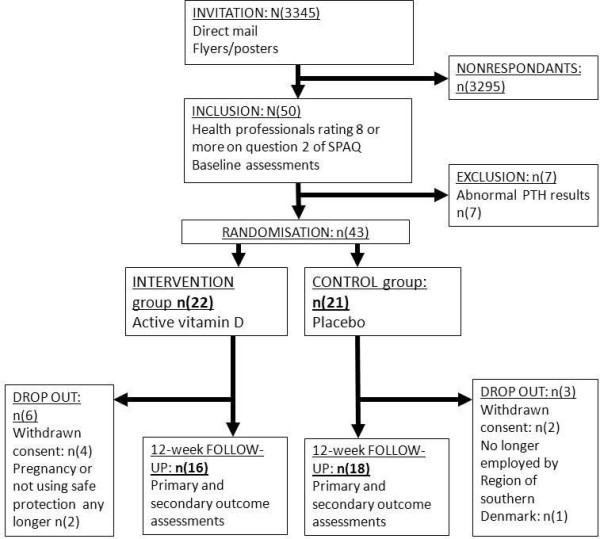
**Flow of participants.**

In total, 3345 healthcare professionals were employed in the two hospitals during the inclusion period. All the employees were invited to participate in the study. 50 were screened by the research nurse, and blood samples were collected during autumn-winter (October, November and December 2011). Before randomisation, 7 were excluded due to abnormal blood samples, mainly because of PTH levels above 9.2 nmol/L. Therefore, 43 participants were included in the trial. The mean SPAQ score was 11.1, and 19 had a SPAQ score below 11. The mean 25(OH)D in excluded employees was lower (mean = 50.9 nmol/L (SD 25.7) than in the participants who completed the study (mean = 68.3) nmol/L (SD 25.3)). Of the 43 participants randomised, 22 received 70 μg of active vitamin D, and 21 received placebo. 34 completed the study, and data were collected for the primary, secondary and exploratory outcomes during winter-spring (January, February, March 2012) (Figure 1).

Drop-out analysis revealed no significant differences between the two treatment groups with respect to completion rates. However, the dropout analysis revealed a selective dropout for participants with low 25(OH)D (mean = 52.7 nmol/L (SD 17.7)) (p = 0.04).

### Demographic data

The demographic and clinical characteristics at baseline were similar in the two groups (p > 0.05 for all) (Table 1). The majority of participants were women (vitamin D group: 100%; placebo group: 90%), with an average age of 48 (SD = 12) in the vitamin D group and 44 (SD = 9) in the placebo group. The majority of participants in both groups had normal 25(OH)D at baseline (Table 1). At endpoint, 7 participants had an insufficient 25(OH)D and 3 had a deficient 25(OH)D, all in the control group. The mean 25(OH)D at 12 weeks was 46.2 (SD = 21.7) in the control group.

**Table 1:** **Demographic and clinical characteristics of participants at baseline**

	Intervention group n = 22	Control group n = 21	Both groups n = 43
Female, n (%)	16 (100)	16 (89)	32 (94)
Age mean (SD), years	44.2 (11.5)	44.4 (10.0)	44.3 (10.6)
Smokers, n (%)	5 (31)	3 (17)	8 (24)
PTH mean (SD), nmol/l	6.1 (1.8)	5.6 (2.0)	5.9 (1.9)
Skin pigmentation mean (SD)	3.0 (1.0)	2.7 (1.1)	2.9 (1.0)
Absenteeism mean (SD), days	1.5 (2.7)	0.6 (1.2)	1.0 (2.0)
Waist circumference mean (SD), cm	90.3 (12.7)	82.5 (8.0)	86.1 (11.0)
Normal 25(OH)D (>50), n (%)	15 (68)	16 (76)	31 (72)
Insufficient 25(OH)D (25–50), n (%)	7 (32)	5 (24)	12 (28)
Deficient 25(OH)D (<25), n (%)	0 (0)	0 (0)	0 (0)

### Outcomes

**Figure 2:**
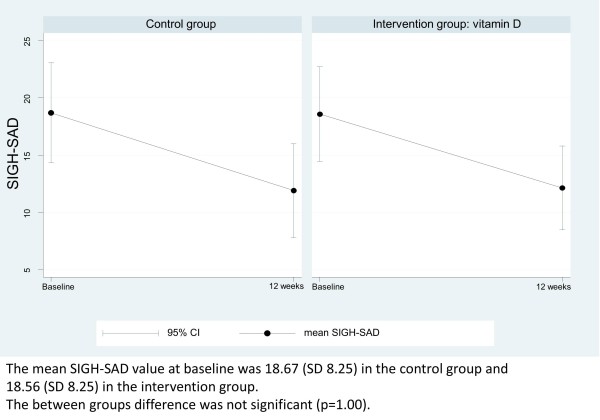
**Depression symptoms on SIGH-SAD.**

There was no significant between-group difference in the decrease of the primary outcome SIGH-SAD over time (intervention group, mean decrease (SD): -6.4 (7.3); control group, mean decrease (SD): -6.8 (9.5); p = 0.7). Baseline values for SIGH-SAD were not significantly different between the intervention group (18.7 (SD 9.3)) and the control group (18.6 (SD 8.3)). The non-significant mean difference in primary outcome was 0.34 in favour of the placebo group. However there was an improvement in SIGH-SAD from baseline to completion of the study for both treatment groups (Figure 2).

There were no significant between-group differences in the secondary outcomes, .i.e. WHO-5 and the exploratory outcomes weight, waist circumference, blood pressure and absenteeism (Table 2).

**Table 2:** **Increase in secondary and explorative outcomes in intervention group (active vitamin D) and control group (placebo)**

	Intervention group n = 22	Control group n = 21	p-value
WHO-5	6.8 (19.8)	13.0 (19.6)	0.42
Weight, kg	0.9 (1.8)	0.6 (1.7)	0.76
Waist circumference, cm	-0.3 (2.9)	-1.4 (4.4)	0.50
Blood pressure, mmHg			
- Systolic	5.0 (13.0)	-0.8 (11.1)	0.21
- Diastolic	1.6 (6.7)	0.3 (9.0)	0.55
Absenteeism, days	1.7 (5.9)	2.6 (5.7)	0.99

Mean increase (SD) from baseline to 12 weeks outcome measurement.

The results according to the second objective revealed a weakly correlation between 25(OH)D and WHO-5 (Spearman rho = 0.20) but no correlated between 25(OH)D and SIGH-SAD (Spearman rho = -0.01) at baseline.

One participant in the control group and none in the intervention group had used nutritional supplements including vitamin D. The results were not affected when correcting for this. The overall treatment adherence was high (85.9 (SD 10.3)). However, two participants with normal 25(OH)D at baseline in the intervention group had the same serum levels at completion. The rest of the intervention group had significantly higher 25(OH)D.

### Side effects and adverse events

There were no significant between-group differences in recorded known side effects (fatigue, muscle spasm, pain, nausea, constipation) or in the number of other adverse events recorded. None of the other adverse events were related to vitamin D. Additionally, between-group differences in serum phosphate and serum calcium were non-significant.

## Discussion

The objective of this study was to investigate whether employees with indoor work and SAD symptoms would benefit from vitamin D supplementation during the winter season, i.e. whether vitamin D would alleviate symptoms of depression during the winter and early spring. The study is qualified due to the block randomisation, which is crucial when including participants during the wintertime. Additionally, the double-blinded randomised design is the best suited to demonstrate an effect of the intervention. Finally, the trial included both psychometric assessments as well as levels of the active treatment. However the design did not favour inclusion of participants with lower 25(OH)D, thus the study did not allow an investigation of the ability of vitamin D to improve mood in those with low 25(OH)D at baseline. Thus, the overall most significant finding in this study was that vitamin D had no effect on SAD symptoms.

The findings are different from those achieved in previous small studies [9, 10] and a larger Norwegian study [12]. In the small studies, the inclusion criterion was 25(OH)D below 40 nmol/L [9, 10], and in the Norwegian study [12], mean baseline 25(OH)D was 52.5 nmol/L. This indicates that vitamin D supplementation might only be relevant for those with lower 25(OH)D. We chose a SPAQ-SAD cut-off at 8 in order to include more participants with SAD symptoms, thus we included participants with less severe symptoms than recommended by Kasper et al. [22]. The lower SPAQ-SAD cut-off might have influenced the results negatively due to a reduced effect on participants with limited symptoms according to the low sensitivity in SPAQ-SAD [28]. The magnitude of this effect is unknown since we did not have scores from the summer months.

Both weight and waist-circumference among the participants were measured since seasonal affective disorder is related to an increased appetite, which again results in an increased risk of the metabolic syndrome. We did not find any improvement in the blood pressure or a positive effect on weight. These findings are in accordance with two other trials which showed no effect of vitamin D supplementation on weight loss among obese patients [29].

There were five main limitations of the study [1]. High PTH was an exclusion criterion for participation. High PTH is correlated with low 25(OH)D, and seven participants were excluded for this reason. This limitation was defined by the legislative authorities because of the risk of placebo treatment of secondary hyperparathyroidism [2]. The drop-outs had a significantly lower 25(OH)D, which reduced the possibility to demonstrate an effect of the intervention [3]. We did not reach the calculated sample size. Thus, the results from the study might have been subject to a type II error. The study was grossly underpowered in a way that detection of clinically significantly results was unlikely. We had expected more participants to enter the study; the most prominent factor for the women was the criterion that anti-conception be practised, which many could not meet. The smaller number of participants, especially among young women, might weaken the generalizability of the study. This apart, we consider these data as being potentially relevant for other staff categories with indoor work [4]. The study was carried out using posters and direct e-mails to the staff at the two hospitals. Thus, we do not know whether the participants were representative of people with SAD symptoms [5]. Finally, we included participants according to their scores on a single question in the SPAQ-SAD rather than the full definition of SAD, but all participants except four had abnormal SIGH-SADs at baseline. Considering this limitation, we did not find a difference between the vitamin D and placebo group but a significant decline of SIGH-SAD in both groups, which could be due to placebo respond. We could not explain it by the duration of daylight since time from solstice did not have an impact on SIGH-SAD.

The participants were respondents of announcements: neither we do not know the prevalence of winter depression among health care professionals, nor we do not know whether the participants had more or fewer symptoms than those who did not respond, reducing the external validity of the study. Additionally the trial might only find relevance for those with milder symptoms and declined by those with more severe symptoms.

The decision to include participants with 25(OH)D between 10–160 nmol/L was taken not only to study the relation between low 25(OH)D status and symptoms of depression but also to test whether a decrease in 25(OH)D during the winter season could be a risk factor for depressive symptoms in those who had previously experienced these symptoms. However, we did not find the anticipated prevalent low 25(OH)D during the wintertime in this study, where those participants thereby could benefit from vitamin D supplementation, which could be explained by the high mean status at baseline.

The study had a clinical perspective focusing on participants who had mild to more severe symptoms of SAD. Unfortunately, we did not add a healthy control group, which could have had a more public health perspective.

The participants were allowed to use LT, and in the written information they were informed that LT has shown a positive effect on SAD. However, only 4 participants used LT, which might be the explanation of no confounder effect on any outcome.

The study does not exclude a positive effect of vitamin D supplementation on SAD, especially in patients with low 25(OH)D. Further investigation of SAD and vitamin D supplementation must therefore include participants with low 25(OH)D. Moreover, studies must be concluded during the early winter months in order to ensure no influence of the increased sunlight after the winter solstice.

## Conclusion

There were no significant between-group differences in the primary (SIGH-SAD) and secondary (WH0-5) as well as the exploratory outcome measures (weight, waist circumference, blood pressure, absenteeism from work and 25(OH)D). Thus, the study failed to demonstrate an effect of vitamin D on SAD symptoms. These finding may be limited by the fact that the study was underpowered, and the inclusion did not favour participants with low 25(OH)D at baseline.

## Abbreviations

25(OH)D: Serum 25-hydroxyvitamin D level
CRP: C-reactive protein (CRP)
eGFR: Estimated glomerular filtration rate
SAD: Seasonal affective disorder
HCG: Human chorionic gonadotropine
LT: Light therapy
PTH: Parathyroid hormone
SIGH-SAD: Structured Interview Guide for the Hamilton Depression Rating Scale, Seasonal Affective Disorders
SPAQ-SAD: Seasonal Pattern Assessment Questionnaire
UVB: Ultraviolet B shortwave
WHO5: World Health Organization-Five Well-Being Index.
